# Supplementary material for: Predicting visual function by interpreting a neuronal wiring diagram
Source: Nature. 2024 Oct 2;634(8032):113–23. doi: 10.1038/s41586-024-07953-5 (PMC11446822; doi:10.1038/s41586-024-07953-5)
Supplement: Supplementary file 6 — CRF and ERF predictions for individual TmY4 and TmY9 cells. Analogous to Supplementary Data 3, but for TmY target types. Shown are the top four monosynaptic pathways, the strongest pathway passing through each of the top ten intermediary types (ranking from Extended Data Fig. 7), and the trisynaptic pathway Tm1–TmY–Dm3–TmY (see the section entitled Prediction of spatial normalization). [file 41586_2024_7953_MOESM6_ESM.zip › DataS4/TmY9q/720575940607579266.pdf]

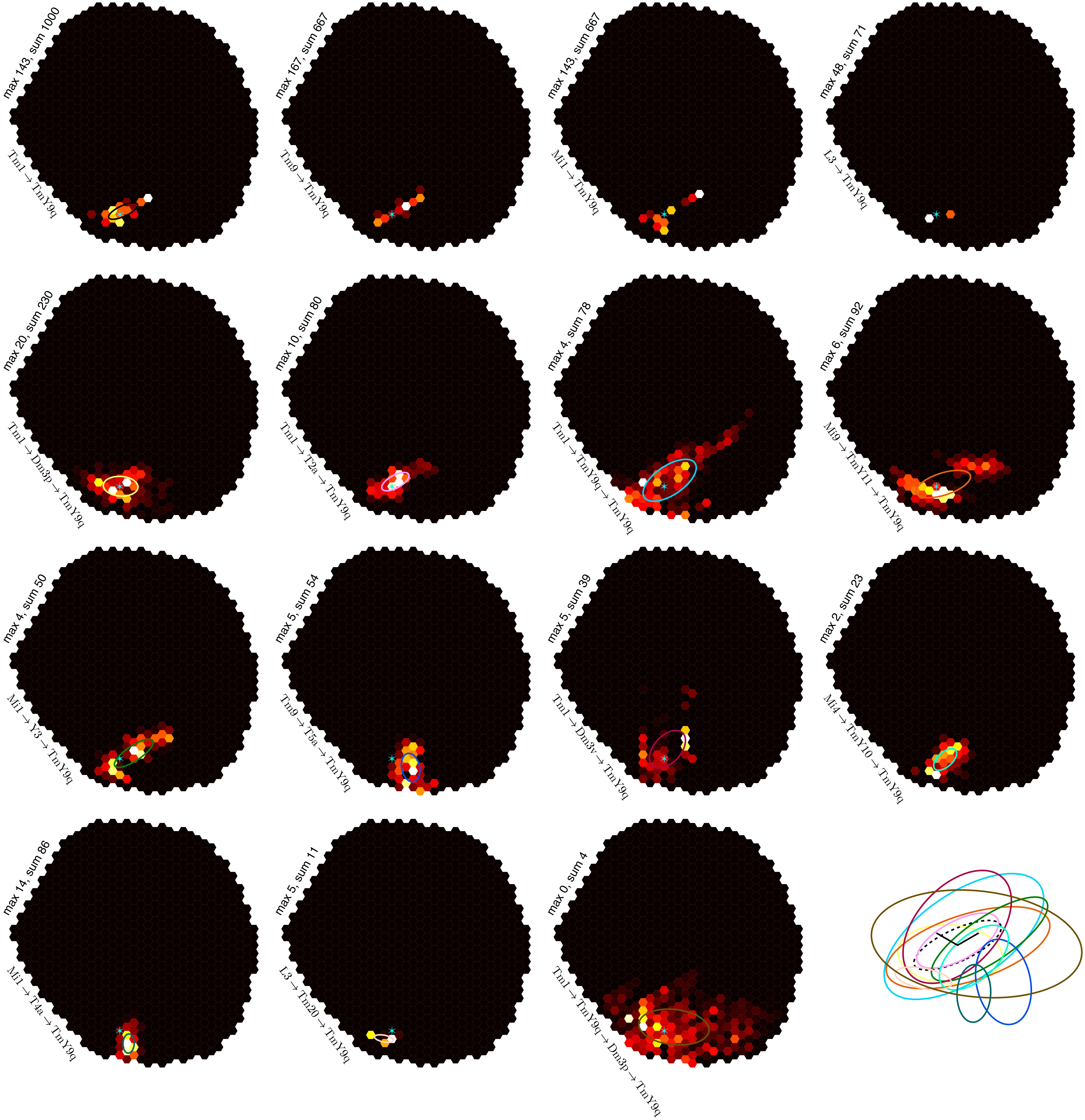
max 143, sum 1000
Tm1 → TmY9q
max 167, sum 667
Tm9 → TmY9q
max 143, sum 667
Mi1 → TmY9q
max 48, sum 71
L3 → TmY9q
max 20, sum 230
Tm1 → Dm3p → TmY9q
max 10, sum 80
Tm1 → T2a → TmY9q
max 4, sum 78
Tm1 → TmY9q → TmY9q
max 6, sum 92
Mi9 → TmY11 → TmY9q
max 4, sum 50
Mi1 → Y3 → TmY9q
max 5, sum 54
Tm9 → T5a → TmY9q
max 5, sum 39
Tm1 → Dm3v → TmY9q
max 2, sum 23
Mi4 → TmY10 → TmY9q
max 14, sum 86
Mi1 → T4a → TmY9q
max 5, sum 11
L3 → Tm20 → TmY9q
max 0, sum 4
Tm1 → TmY9q → Dm3p → TmY9q
